# Dr. Knopf on Consumption

**Published:** 1908-06

**Authors:** 


### DR. KNOPF ON CONSUMPTION.

Dr. S. A. Knopf, of New York, delivered an exceelingly interesting and instructive public lecture at the Grand Opera House, April 26th, under the auspices of the Atlanta Sanitary and Tuberculosis Prevention Society.

The importance of preventing tuberculosis is only known to physicians and a few of the laity and such lectures by men who have given the question so much thought and study must eventually be of great value in lessening the great white plague, or as Dr. Todd aptly calls it in the South "the great black plague."

There are few bits of medical literature that have received wider circulation and more appreciation that has Dr. Knopf's prize essay on the prevention of tuberculosis. The amount of good it has done cannot be easily estimated.

Dr. Knopf also appeared before the Fulton County Medical society and made address to the physicians of Atlanta. His thorough familiarity with all phases of the subject and his attractive manner of presenting it make him a most interesting speaker.

